# MRCKα Is Dispensable for Breast Cancer Development in the MMTV-PyMT Model

**DOI:** 10.3390/cells10040942

**Published:** 2021-04-19

**Authors:** Mei Qi Kwa, Rafael Brandao, Trong H. Phung, Jianfeng Ge, Giuseppe Scieri, Cord Brakebusch

**Affiliations:** 1 Biotech Research and Innovation Center (BRIC), University of Copenhagen, Ole Maaløes vej 5, 2200 Copenhagen, Denmark; Kwa_Mei_Qi@sris.a-star.edu.sg (M.Q.K.); rafael.brandao@bric.ku.dk (R.B.); tphung@som.umaryland.edu (T.H.P.); gejianfeng1987@yahoo.com (J.G.); scieri.giuseppe@gmail.com (G.S.); 2 Centre College, 600 W Walnut St, Danville, KY 40422, USA; 3 Medical Research Centre (MRC) Cancer Unit, Hutchison/MRC Research Centre, University of Cambridge, P.O. Box 197, Biomedical Campus, Cambridge CB2 0XZ, UK

**Keywords:** MRCK, breast cancer, invasion

## Abstract

MRCKα is a ubiquitously expressed serine/threonine kinase involved in cell contraction and F-actin turnover, which is highly amplified in human breast cancer and part of a gene expression signature for bad prognosis. Nothing is known about the in vivo function of MRCKα. To explore MRCKα function in development and in breast cancer, we generated mice lacking a functional MRCKα gene. Mice were born close to the Mendelian ratio and showed no obvious phenotype including a normal mammary gland formation. Assessing breast cancer development using the transgenic MMTV-PyMT mouse model, loss of MRCKα did not affect tumor onset, tumor growth and metastasis formation. Deleting MRCKα and its related family member MRCKβ in two triple-negative breast cancer cell lines resulted in reduced invasion of MDA-MB-231 cells, but did not affect migration of 4T1 cells. Further genomic analysis of human breast cancers revealed that MRCKα is frequently co-amplified with the oncogenes ARID4B and AKT3 which might contribute to the prognostic value of MRCKα expression. Collectively, these data suggest that MRCKα might be a prognostic marker for breast cancer, but probably of limited functional importance.

## 1. Introduction

Rho GTPases are small GTPases regulating the organization of the actin cytoskeleton and cell migration, but also differentiation, proliferation and other cellular processes [1]. They exert their function by binding and activating in their GTP bound form to effector molecules which then mediate the biological effects. The myotonic dystrophy kinase-related Cdc42-binding kinase α (MRCKα; gene name *CDC42BPA*) is an effector of the ubiquitously expressed Rho GTPases Cdc42 and possibly Rac1 [2,3]. Binding of Cdc42 to MRCKα leads to autophosphorylation of MRCKα at S1003, which correlates with kinase activity [4]. Kinase activity is furthermore regulated by diacylglycerol binding to the centrally located C1 domain [5]. The MRCKα kinase domain is about 80% homologous to the kinase domain of MRCKβ [6,7,8], and furthermore similar to the kinase domain of ROCK1/2, two closely related Rho effectors. This is believed to result in a shared set of substrates between MRCKα, MRCKβ and ROCK1/2, including the regulatory subunits of the myosin light chain phosphatase (MYPT1) and of myosin II (MLC2) and the LIM kinases 1 and 2 [9]. Since phosphorylation of MYPT1 and MLC2 promotes cell contraction, while phosphorylation of the LIM kinases mediates inactivation of the F-actin severing cofilin, MRCKα is proposed to play an important role in the regulation of cell migration in collaboration with MRCKβ, ROCK1 and ROCK2.

Indeed, several studies support an important role for MRCKα in cancer cell migration. In elongated MDA-MB-231 breast cancer cells, contraction and invasion into collagen or matrigel is jointly regulated by MRCKα, MRCKβ, ROCK1 and ROCK2 [9,10]. Inhibition of either MRCKα/β or ROCK1/2 alone had only a partial effect. Invasion of the squamous cell carcinoma line SSC12 in an organotypic skin culture model, on the other hand, was largely dependent on MRCKα and MRCKβ function [11]. In 2D migration, inhibition of both MRCKs by BDP9066 significantly reduced migration speed without affecting directionality [4]. However, MRCKs and ROCKs do not appear to always have overlapping effects. In neurite outgrowth assays in vitro, MRCKα is required for outgrowth, while ROCK1 promotes retraction [12]. Additionally, MRCKα/β are responsible for promoting SCC cell invasion through the matrix, whilst ROCK1/2 signaling contributes only in the leading fibroblasts to migration [11].

Little is known about the in vivo function of MRCKα during development and in disease, but several studies indicated a role for MRCKα in tumor formation. In DMBA/TPA induced skin tumor in mice, a strongly increased expression of MRCKα and MRCKβ was observed [4]. Treatment with the MRCK inhibitor BDP9066 reduced the total tumor volume, but not the frequency of the papilloma, suggesting a role for MRCKs in tumor growth. This notion was supported by antiproliferative effects of BDP9066 on many cancer cell lines in vitro. In human breast cancer, MRCKα copy number was reported to be increased in 64% of 852 breast cancer samples at the Wellcome Trust Sanger Institute Cancer Genome Project [8]. Moreover, in a gene expression study of breast cancer patients, MRCKα, alias PK428, was identified as part of a profile of 70 genes predicting short interval to metastasis formation and poor prognosis [13]. These data suggest a potential role for MRCKα in breast cancer, although no validation analysis has been carried out.

To directly address the role of MRCKα in malignant breast cancer, we generated mice with a constitutive knockout of MRCKα using CRISPR genome editing and investigated their development and, by crossing with MMTV-PyMT mice, the formation of breast cancer. In addition, we tested redundancy of MRCKα and MRCKβ in breast cancer cell lines in vitro. Our data reveal a dispensable role in MMTV-PyMT breast cancer model and cell type specific roles in 3D collagen gel invasion of breast cancer cells. In addition, we uncovered a co-amplification of MRCKα with two other known oncogenes in human cancers, which might contribute to the worse prognosis of breast cancer patients with amplified MCRCKα.

## 2. Materials and Methods

### 2.1. Generation of MRCKα Ko Mice and Mammary Cancer Model

MRCKα KO mice were generated by the Transgenic Mouse Core Facility, University of Copenhagen, by direct injection of Cas9 mRNA and a guide RNA targeting exon 1 of mouse MRCKα (5′–TGTCCGGAGAAGTGCGTTTG AGG) to zygotes. The PAM sequence is underlined. Injected zygotes were embryo-transferred and mice born were genotyped by sequencing of the amplified target region. KO mice investigated in this study were genotyped with following a touchdown PCR protocol. The PCR cycling conditions were: 95 °C for 3 min, a touchdown phase consisting of 10 cycles of 95 °C for 30 s, a temperature ramp from 63 °C to 58 °C for 45 s at 0.5 °C per cycle, 72 °C for 1 min and then a second phase consisting of 25 cycles of 95 °C for 30 s, 58 °C for 45 s, 72 °C for 1 min and a final extension step of 72 °C for 10 min.

For the spontaneous mammary tumour model, MMTV-PyMT MRCKα HET male mice were crossed with HET female. MMTV-PyMT HET and MMTV-PyMT KO mice were monitored daily and sacrificed once the total tumour volume reached 1000 mm^3^. All animal experiments were carried out following national and European animal welfare regulations and were approved by the Danish Board for Animal Experiments (2016-15-0201-00946).

### 2.2. Quantification of Lung Micrometastasis

The number of micrometastases was counted manually on fixed lungs by Hematoxylin-Eosin staining of 5 μm paraffin sections of the left lung. Three random sections at least 100 μm apart in distance in the tissue were used for quantification per sample.

### 2.3. Primary Mammary Tumor Organoids

Primary tumor organoids were isolated from mammary tumors as previously described [14,15]. Briefly, mammary tumors were harvested from mice reaching end-point, minced with a scalpel and digested for 1 h at 37 °C in a collagenase solution (DMEM/F12 containing 2 mg/mL collagenase, 2 mg/mL trypsin, 5% FBS, 5 μg/mL insulin and 50 μg/mL gentamicin). The suspension was centrifuged at 500× *g* for 10 min and the supernatant was carefully removed. The pellet was resuspended in warm DMEM/F12 media containing 1 U/μL DNAse I and incubated for 5 min. The suspension was then filtered through a 100 μm cell strainer and then pulse-centrifuged at 500× *g* to remove the supernatant which contains fibroblasts. The pulse-centrifuging step was repeated a total of 4 times, or until the supernatant runs clear. The final pellet was resuspended in organoid culture media (DMEM/F12 containing 1% penicillin/streptomycin, 1% insulin-transferrin selenium and 2.5 nM human fibroblast growth factor basic) prior to being embedded in collagen I matrix. 

### 2.4. Carmine Alum and Nissl Staining 

For mammary gland branching analysis, 4th inguinal mammary fat pads were prepared as whole mount samples and carmine alum staining was performed according to the protocol as previously described [16]. For the analysis of brain sections, sagittal brain sections at 5 μm thickness were stained in 0.5% cresyl violet solution containing 3% glacial acetic acid, according to standard procedures for Nissl staining. 

### 2.5. Cell Lines

Human MDA-MB-231 and murine 4T1 cells (both obtained by J. Erler, BRIC, University of Copenhagen). Immortalized mouse cancer associated fibroblasts were isolated from wildtype MMTV-PyMT tumors and immortalized by retroviral transduction of HPV-E6 29 [17]. CAFs were cultured in low- glucose DMEM containing 10% fetal bovine serum, 1% penicillin/streptomycin (Thermo Fisher Scientific, Lillerød, Denmark and 1% Insulin-Transferrin-Selenium (ITS-G) from Gibco. Cells were treated as indicated with 10 μM Y-27632 or 1 μM latrunculin (both Tocris, Abingdon, UK). All cell cultures were maintained at 37 °C, 5% CO_2_. 

### 2.6. Lentiviral Transduction

Single guide RNAs (sgRNA) targeting human MRCKα and MRCKβ coding regions were designed and cloned into the lentiviral vector lentiCRISPRv2 (GeCKO; Addgene, Watertown, MA, USA) according to manufacturer’s instructions. The oligonucleotide sequences corresponding to the human sgRNAs used were:

hMRCKa guide 1: F: 5′ CACCGGCGGGCCCGCTCAGACCAA, R:5′ AAACTTGGTCTGAGCGGGCCCGCC
hMRCKa guide 2 F: 5′ CACCGGAAGTGCGTTTGAGGCAGT, R:5′ AAACACTGCCTCAAACGCACTTCC
hMRCKb guide 1 F: 5′ CACCGGGCGGCACCATGTCGGCCA, R:5′ AAACTGGCCGACATGGTGCCGCCC
hMRCKb guide 2 F: 5′ CACCAGGGCGCTCTCGTTGCGCCA, R:5′ AAACTGGCGCAACGAGAGCGCCCT

The oligonucleotide sequences corresponding to the mouse sgRNAs used were:

mMRCKa guide 1: F: 5′ CACCGGGTCCGGAGAAGTGCGTTTG, R:5′ AAACCAAACGCACTTCTCCGGACCC
mMRCKa guide 2 F: 5′ CACCGGAAGTGCGTTTGAGGCAGT, R:5′ AAACACTGCCTCAAACGCACTTCC
hMRCKb guide 1 F: 5′ CACCGGGGGCGGAGTTCCTCGAGTG, R:5′ AAACCACTCGAGGAACTCCGCCCCC

Since no obvious differences were observed between ko cells generated with guide 1 or guide 2, results obtained with different guides for the same gene were pooled.

To produce viral particles, HEK293T cells were transfected with lentiviral vectors following standard procedures and viral supernatant was used to infect target cell lines. Selection was done using either puromycin (1–2 μg/mL) or blasticidin (8–10 μg/mL), 48 h post-transduction. An empty vector was transduced into cells as control.

### 2.7. Cell Adhesion

A 96-well tissue culture plate was coated for 1 h at room temperature using type I rat tail collagen serially diluted in PBS starting from 10 μg/mL to 0.00064 μg/mL. After coating, the plate was washed three times with PBS and then blocked in 1% BSA for 1 h. The plate was washed again for three times in PBS and cells are then seeded in serum-free media at a density of 2 × 10^4^ cells per well and allowed to adhere. After 30 min of incubation at 37 °C, the plate was washed one time with PBS and the cells were given complete media and incubated at 37 °C for 3 h. The cells were fixed in 4% PFA for 20 min at room temperature and then stained in crystal violet diluted in 20% methanol for 15 min. After washing in PBS and then water, the plate was dried and the stain was extracted by 1% SDS in PBS. The extracted dye was transferred to a 96 well plate and absorbance at 560 nm was then read from the plate. 

### 2.8. Cell Proliferation Assay

Cells were seeded at a density of 7 × 10^3^ per well in a 12 well plate format. After overnight incubation, the cells were placed in the Incucyte system for live cell imaging and automated quantification. An image was taken every 2 h, for a total of 48 h.

### 2.9. F and G Actin Fractionation

For fractionation of the F and G actin cells were lysed in actin stabilization lysis buffer (50 mM PIPES, 50 mM NaCl, 5 mM MgCl_2_, 5 mM EGTA, 2 mM ATP, 5% glycerol, 0.1% Nonidet P-40, 0.1% Triton X-100, 0.1% Tween 20, 0.1% β-mercaptoethanol, protease inhibitors and phosphatase inhibitors) for 10 min at 37 °C. Cell debris were removed by centrifugation at 300× *g* and protein concentration of the supernatant was determined. After equilibrating the protein concentration of the samples using the lysis buffer, an aliquot of the lysate (10% of total volume) was stored as total protein input, while the remainder was subjected to ultracentrifugation at 100,000× *g* for 1 h at 37 °C. After centrifugation, the supernatant containing G actin was carefully transferred to a new tube and stored, while the pellet containing F actin was resuspended in cold lysis buffer containing 1 μM cytochalasin D and incubated on ice for 45 min to dissolve the fibers into monomers. All fractions were then boiled in Laemmli buffer and prepared for Western blotting.

### 2.10. Western Blot Analysis

Tissue fragments and cultured cells were lysed with Laemmli buffer. Lysates were separated by a 10% SDS-PAGE and Western blotting was carried out following standard protocols. For detection, the following primary antibodies were used: MRCKα (#374568), MRCKβ (#374597), GAPDH (#25778; all SantaCruz Biotechnology, Dallas, TX, USA), pMLC (#3674), pCofilin (#3311; all Cell Signaling, Danvers, MA, USA), β actin (#AB6276, Abcam, Cambridge, MA, USA). For detection, appropriate HRP coupled secondary antibodies were used: horse-anti-mouse IgG (#VECTPI2000), goat-anti-rabbit IgG (#VECTPI1000, all Vector Laboratories, Burlingame, CA, USA). LuminataTM Western HRP Chemiluminescence Substrates detection reagent (Milipore, Hellerup, Denmark) was used for chemiluminescence, which was then detected with Medical X-ray film (AGFA, Birkerød, Denmark). The intensity of the bands was quantified using ImageJ software.

### 2.11. D Spheroid Invasion Assay 

Spheroids were generated from aggregated cells, either homogenously or heterogeneously in combination with CAF [17]. Briefly, 500 cancer cells (with or without 500 CAFs) were seeded into 96 well round bottom plates containing 20% methylcellulose in serum-free DMEM. After overnight incubation at 37 °C and 5% CO_2_, the aggregated spheroids were embedded in 2 mg/mL collagen I (Corning, Glendale, AZ, USA) in 12-well plates at approximately 10 spheroids per well. After polymerization, the collagen matrix was overlaid with 500 μL of DMEM containing 2% FBS and, when indicated, 10 μg/mL Y-27632 (Sigma Aldrich, St. Louis, MO, USA). The spheroids were imaged immediately after overlaying with media to obtain the initial area of the core spheroid and again after 24 h incubation. Phase contrast images were captured using an Olympus CellSens imaging software. 

### 2.12. Spheroid Image Quantification

For quantification, images were analyzed using Fiji/ImageJ [18,19]. Spheroid area measurements were determined semi-automatically using a custom macro on ImageJ. First, images were smoothed by applying a Gaussian blur filter with a sigma of 2. Next, the spheroid was outlined by manual thresholding. Gaps in the spheroids were filled with the Fill Hole command and the total area of the spheroid was then measured. The area of the spheroid imaged at 24 h post-embedding was subtracted from that obtained at 0 h, to obtain the final area of the invasion zone.

### 2.13. Time-Lapse Microscopy

5 × 10^3^ MDA-MB-231 cells stably expressing a LifeACT-GFP plasmid (Addgene, Watertown, MA, USA) were seeded in 8-well chambered glass slides. 24 h post-incubation, the cells were then imaged by time-lapsed microscopy on a DeltaVision suite Olympus microscope. Images were captured every 5 s for a total of 10 min, under a 40× magnification using immersion oil. Kymographs were generated as described previously using ImageJ [20]. Briefly, a line was first drawn from the middle of the cell perpendicularly towards the migrating front of cells. Then, the Reslice function was used to generate the kymograph. A new line was again drawn along each actin protrusion on the kymograph and the enclosed triangle, of which each dimension corresponds to the protrusion length (P), persistence (S) and protrusion rate (P/S), were then measured in pixel units. All lines are indicated in the corresponding figure.

### 2.14. RNA-Sequencing and Data Analysis

Total RNA was isolated from vector control, MRCKα KO, MRCKβ KO or DKO MDA-MB-231 cells using standard RNA purification kit (Sigma-Aldrich, St. Louis, MO, USA). For DKO samples, 2 different sets were generated using the guide 1 or the guide 2 gRNAs for hMRCKα or hMRCKβ. Two sets of control samples were obtained from two separate transductions of the vector plasmid. RNA sequencing was performed by the Beijing Genomics Institute (BGI). Sequencing depth was at least 10 M clean reads per sample via SE50. To compare the differential gene expression, the FPKM values obtained from the analysis performed by BGI were used. For the control and DKO samples, FPKM values from both transductions (vector) or both gRNA sets (DKO) were first compared and FPKM of genes that differed by more than 2-fold were excluded from subsequent analysis. An average is obtained from the remaining genes. Log2 fold-change was then calculated for the MRCKα KO, MRCKα KO and DKO samples against vector control. An arbitrary log2 fold change of 1 (and −1) was used as threshold for differential expression.

### 2.15. Statistics

Data are presented as means with error bars representing standard error of the mean. Statistical significance was determined by the two-tailed Student’s *t*-test, if not indicated otherwise. For all statistical analyses, *p* values are denoted as: *** *p* < 0.001, ** *p* < 0.01 and * *p* < 0.05.

## 3. Results

### 3.1. MRCKα Expression Correlates with Survival in Triple-Negative Breast Cancer 

**Figure 1:**
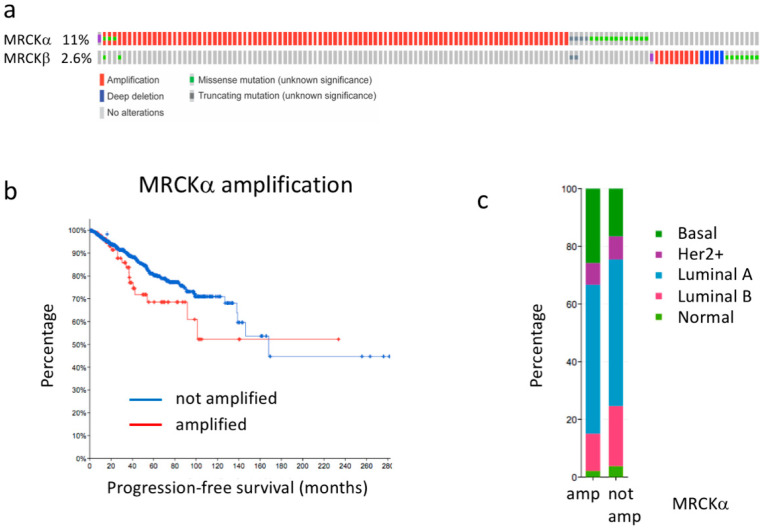
MRCKα gene is amplified in breast cancer. (**a**) Genetic alterations of the MRCKα and MRCKβ genes in breast cancer. (**b**) Progression free survival of breast cancer patients with amplified and non-amplified MRCKα gene (*p* = 0.087). (**c**) Breast cancer subtype distribution of patients with amplified (amp) and not amplified (not amp) MRCKα gene. All graphs were generated in cBioPortal.org using the TCGA PanCancer Atlas data set for invasive breast carcinoma (accessed on 20 March 2021).

MRCKα expression has been reported to be increased in breast cancer. Indeed, around 11% of the breast cancers of the TCGA breast cancer data set (cbioportal.org) [21,22] showed genetic alterations of the MRCKα gene, most of them amplifications (Figure 1a). The closely related MRCKβ gene was only altered in 2.6%, including both amplifications and homozygous deletions (Figure 1a). Further investigating the patients with amplifications of the MRCKα gene, we observed a trend towards reduced progression free survival, suggesting that amplification of the MRCKα gene might be disease relevant (*p* = 0.087; Figure 1b). Amplifications of the MRCKα gene were observed in Basal, Her2+, Luminal A, Luminal B and Normal breast cancers, but were enriched in the Basal subgroup (Figure 1c). Average expression of MRCKα in the amplified subset was increased by about 21% compared to the non-amplified subset (*p* = 0.015). In contrast, increased expression of MRCKβ showed no obvious effect on breast cancer survival and cancers with high expression of MRCKβ were strongly depleted in the Basal subset (Supplementary Figure S1). These data suggest that MRCKα expression might be of relevance for breast cancer formation and progression in all subtypes, but probably most in Basal Cancer.

### 3.2. MRCKα Ko Mice Do Not Show an Obvious Phenotype

**Figure 2:**
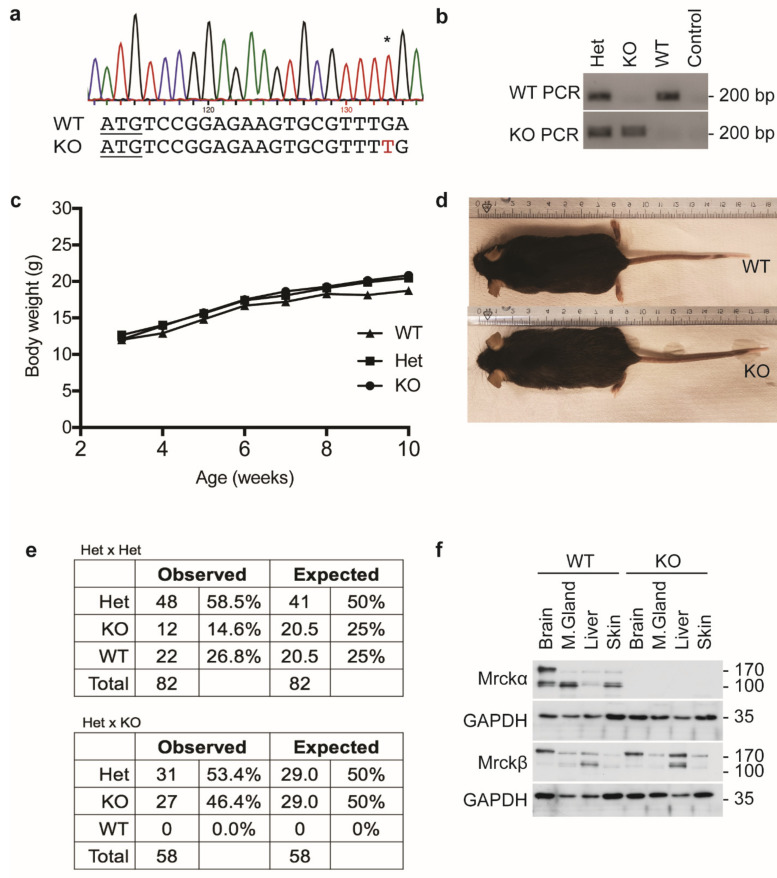
MRCKα ko mice show no obvious phenotype. (**a**) Genotype profile of ko mice showing the p.Leu7fs*35 mutation. * indicates the inserted nucleotide in the chromatogram. (**b**) Representative results from genotyping PCR using primers targeting the mutation specifically. (**c**) Body weight measurements of post-weaning female wild-type, heterozygous and ko mice. (**d**) Representative images of wild-type and ko mice. (**e**) Mendelian ratio of the observed and expected frequencies of wild-type, heterozygous and ko mice in Het × Het mating (*n* = 82) and Het × KO (*n* = 58) mating pairs. (**f**) Immunoblot analysis of MRCKα and MRCKβ expression in wild-type and ko mice.

To analyze the requirement of MRCKα for breast cancer, we first generated mice lacking a functional MRCKα gene (Cdc42bpa) using CRISPR genome editing. Several MRCKα mutant mouse lines were generated and the p.Leu7fs*35 line (KO) in which an extra “T” was introduced into the leucine 7 codon (Figure 2a) was chosen for further analysis.

Homozygous KO mice developed without obvious defects and showed normal size and body weight (Figure 2b–d). Cage behavior (explorative activity, body posture, culling) was indistinguishable from control litter mates (wildtype, heterozygous) and both male and female ko mice were fertile. Crosses of heterozygous mice (Het), showed a slight reduction of KO mice compared to the expected level (Figure 2e). However, checking embryonic litters at different time points before birth no evidence for prenatal lethality or morphological defects could be found (data not shown). Moreover, crosses of KO and Het were born roughly at the Mendelian ratio, giving no indication for partial embryonic lethality (Figure 2e).

To confirm the loss of MRCKα on protein level, lysates of brain, mammary gland, liver and skin were analyzed by Western blotting. WT mice showed that, in addition to the expected isoform of about 190 kD corresponding to the full-length MRCKα, smaller isoforms of about 130 kD were detectable. In brain, larger and smaller isoforms were equally expressed, while in the other tissues the smaller isoform was dominant (Figure 2f). Both isoforms were not detectable in KO mice, indicating efficient gene deletion and specificity of the antibody. The closely related MRCKβ was also expressed in different isoforms in all tissues tested. In addition, liver, the high molecular weight isoform was more prevalent (Figure 2f). Loss of MRCKα did not result in increased protein levels of MRCKβ in the tissues investigated.

**Figure 3:**
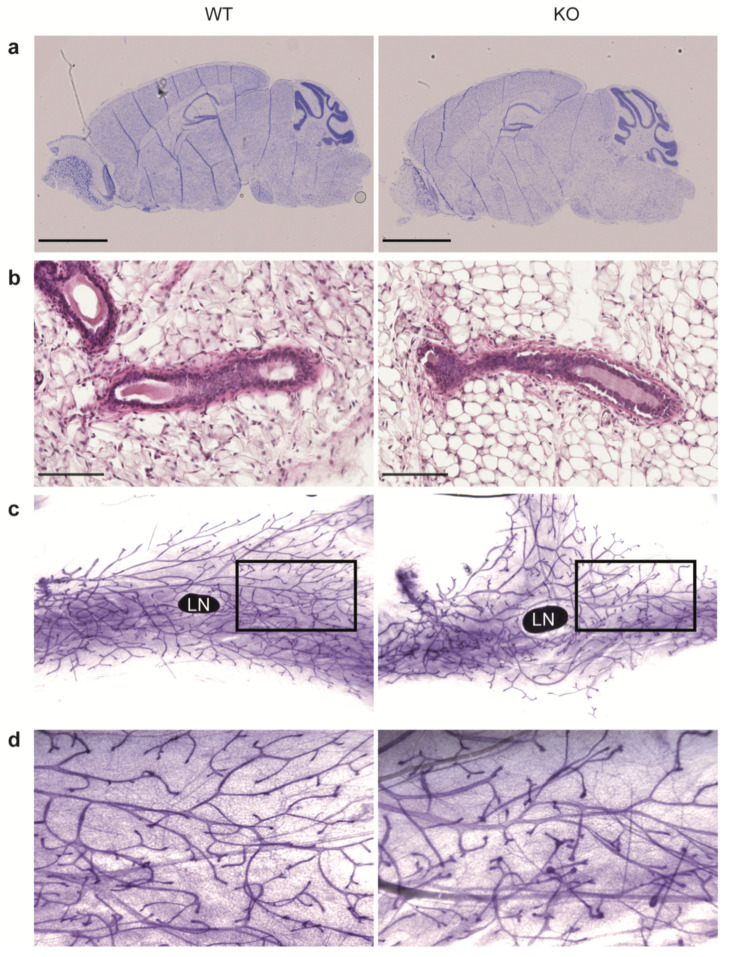
Mrckα knockout does not affect brain and mammary gland development. (**a**) Representative Nissl staining of sagittal brain sections from adult wild-type and ko mice. Scale bars: 2.5 mm. (**b**) Representative H&E staining of the 4th inguinal mammary glands from 7-weeks old virgin female wild-type and KO mice. Scale bars: 100 μm. (**c**) Representative carmine alum-stained mammary gland whole mounts. LN denotes the inguinal lymph node. A black box was drawn to indicate the area of magnification of the image, as shown in (**d**).

Since MRCKα was highly expressed in brain and in mammary gland we assessed both tissues for histological changes in the KO mice. However, Nissl staining of sagittal brain sections (Figure 3a) revealed no obvious alterations. Histological analysis of the mammary gland by hematoxylin/eosin staining indicated normal ducts surrounded by a thin layer of connective tissue in both WT and KO (Figure 3b). Moreover, whole mount staining by carmine alum indicated normal branching morphogenesis of the mammary ducts in MRCKα KO mice (Figure 3c,d).

These data reveal that MRCKα is not required for development. Importantly, the normal development of the mammary gland and the normal overall phenotype provides an excellent basis for the analysis of the role of MRCKα in breast cancer development, since a potential tumor phenotype will not be affected by a developmental phenotype.

### 3.3. MRCKα Is Not Essential for Tumorigenesis or Metastasis in the Mmtv-Pymt Breast Cancer Model

To investigate the role of MRCKα in breast cancer in an in vivo model, we crossed the MRCKα KO mice with MMTV-PyMT mice, which spontaneously develop invasive ductal breast carcinoma with metastases in the lung [23].

**Figure 4:**
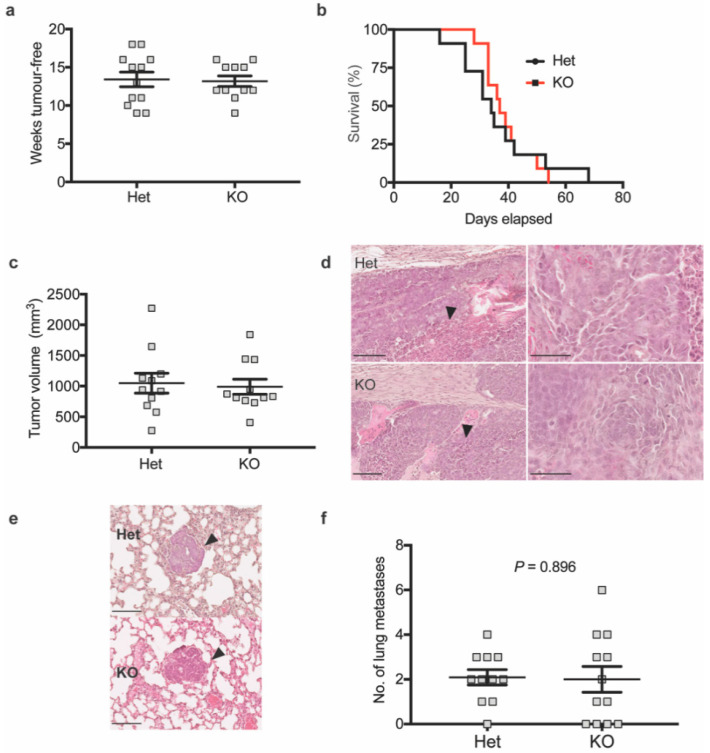
Mrckα knockout does not affect primary mammary tumour development and metastasis in MMTV-PyMT mice. (**a**) Weeks tumour-free (**b**) survival analysis (*p* = 0.8243, log-rank test) and (**c**) tumor volume at end-point of MMTV-PyMT heterozygous (Het) vs. KO mice (*n* ≥ 10/10). (**d**) Representative H&E staining of MMTV-PyMT heterozygous or Mrckα KO mice primary tumours showing extensive necrosis (black arrows). Images on the right represent the same images at higher magnification showing the abnormal cancer cell nuclei. Scale bars: 100 μm. (**e**) Representative H&E staining of lung metastases of MMTV-PyMT heterozygous vs. KO mice. Scale bars: 100 μm. (**f**) Quantitation of lung metastases by counting of foci from the left lung lobe from MMTV-PyMT heterozygous and KO mice upon reaching end point. (*n*: 10/10; mean ± SEM, two-tailed unpaired *t*-test).

Surprisingly, loss of MRCKα did not affect the tumor onset, tumor-free survival and tumor volume (Figure 4a–c). Histological analysis of tumors in KO and Het control mice indicated adenocarcinoma characterized by increased size of nuclei, nuclear pleomorphy, prominent nucleoli and areas of necrosis in more advanced carcinoma (Figure 4d). Occasional infiltration of mononuclear cells and desmoplasia were observed. Tumors appeared relatively solid without single cells or small groups of cells detached from the main tumor. Western blot analysis of tumors confirmed the lack of MRCKα and showed no clear compensatory upregulation of MRCKβ (Supplementary Figure S2a). In one of 6 KO tumors, MRCKα expression was even decreased. Frequency and histology of lung metastases was similar in Het and KO mice (Figure 4e,f).

To identify qualitative changes in the invasion process, we monitored breast cancer invasion in tumor organoids embedded in collagen. Collective cell migration of cancer cells into the collagen gel was detected in both Het and KO cancers without any apparent alterations in KO tumor organoids (Supplementary Figure S1b). These data suggest that MRCKα is not essential for breast cancer development, progression and lung metastasis in the MMTV-PyMT model.

### 3.4. MRCKα Is Important for 3D Collagen Gel Invasion of MDA-MB-231 Breast Cancer Cells

The unaltered breast cancer development in MRCKα ko mice could be related to functional redundancy of MRCKα with MRCKβ. To investigate this possibility, we deleted the genes for MRCKα and MRCKβ individually or alone in two breast cancer cell lines. Since it was shown earlier that siRNA mediated knockdown of MRCKα and MRCKβ together reduces matrigel invasion of the triple-negative breast cancer cell line MDA-MB-231 [9], we chose this cell line for the experiments.

**Figure 5:**
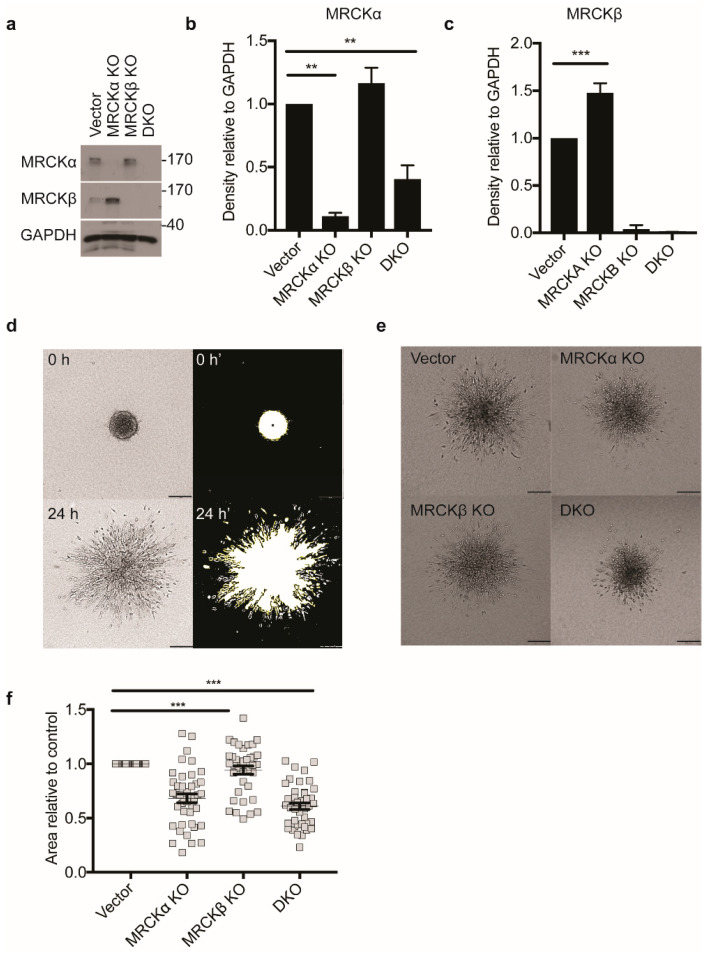
MRCKα and MRCKβ knockout affect 3D invasion. (**a**) Representative immunoblot showing successful MRCKα and MRCKβ, single and double knockout (DKO) in MDA-MB-231 cells. Quantitation of (**b**) MRCKα and (**c**) MRCKβ expression in lysates relative to GAPDH (*n* = 3, mean ± SEM, one-way ANOVA; ** *p* ≤ 0.01; *** *p* ≤ 0.001). (**d**) Strategy for quantifying area of invasion of spheroids by thresholding using ImageJ. Briefly, area of spheroids immediately after embedding in type I collagen was subtracted from the total area of the same spheroids 24 h post-embedding, to obtain the area of invasion (**e**) 3D matrix invasion of vector control, MRCKα KO, MRCKβ KO, or DKO MDA-MB-231 heterospheroids co-cultured with CAFs and embedded in type I collagen. Scale bar: 200 μm. (**f**) Area of collagen invasion relative to vector control (*n* = 3, totaling > than 100 cells per group; mean ± SEM, one-way ANOVA; *** *p* ≤ 0.001).

MDA-MB-231 breast cancer cells lacking MRCKα, MRCKβ, or both (DKO) were established by lentiviral CRISPR genome editing and selection for stably transduced cells. As a control, MDA-MB-231 cells were transduced with the empty vector. Control MDA-MB-231 cells expressed primarily the high molecular weight isoforms of MRCKα and MRCKβ (Figure 5a). Even without clone picking, the efficiency of gene deletion was very high as tested on protein level (Figure 5a–c). Interestingly, KO of MRCKα resulted in a slight but significant increase in MRCKβ expression. On the other hand, loss of MRCKβ did not affect MRCKα protein levels to a significant extent.

Next, invasion of spheroid aggregates of the different MDA-MB-231 breast cancer cell lines mixed with CAFs into 3D collagen gels was assessed. Initially, all spheroids were relatively compact and of similar diameter, although some cells were already detached from the main cell mass at 0 h (Figure 5d; Supplementary Videos S1 and S2). Reproducibly, the number of detached cells at 0 h was lower for DKO than for WT, suggesting reduced invasiveness (Supplementary Videos S1 and S2). For the quantification, only the differences between 24 h and 0 h was taken into consideration (Figure 5d). 

After 24 h, MRCKα KO spheroids showed significantly less invasion than control cells (Figure 5e,f). DKO spheroids also showed defective 3D migration, while MRCKβ KO spheroids were indistinguishable from controls. Movies demonstrated that both control and DKO cells invaded the collagen gel mostly in a rounded and less frequently in an elongated form (Supplementary Videos S1 and S2).

These data indicate a role for MRCKα in the collagen invasion of MDA-MB-231 breast cancer cells, but not for MRCKβ. 

### 3.5. Subtle Cellular and Biochemical Alterations in Mutant MDA-MB-231 Cells Cultured in 2D

**Figure 6:**
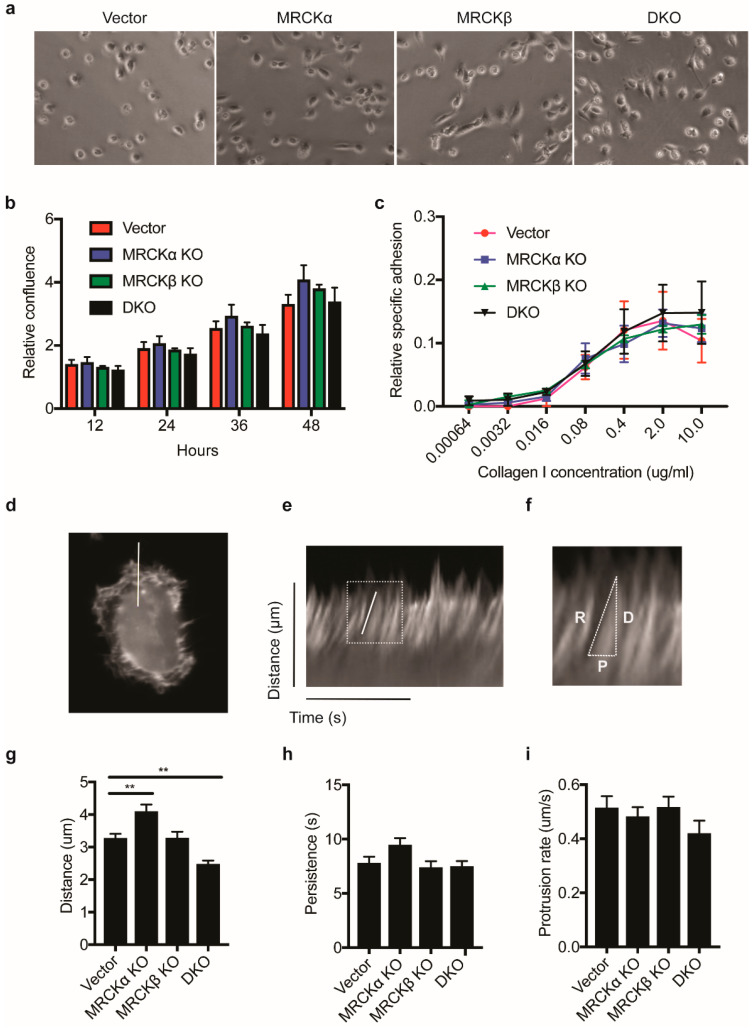
MRCK KO has a modest effect on F-actin polymerization. (**a**) Representative phase contrast images showing the morphologies of control, MRCKα KO, MRCKβ KO, or DKO MDA-MB-231 cells (**b**) Proliferation rate of MRCKα KO, MRCKβ KO and DKO MDA-MB-231 cells as measured by relative confluency over time. (**c**) Relative adhesion of MRCKα KO, MRCKβ KO and DKO MDA-MB-231 cells to increasing concentration of type I collagen. Data is obtained for 3 biological replicates. (**d**–**i**) Kymograph analysis of actin protrusion distance, rate and persistence in MRCK KO cells (**d**) Representative still image of an MDA-MB-231 cell expressing LifeAct-GFP used in live imaging. A white line was drawn perpendicular to the cell body to indicate the position where kymograph was obtained. (**e**) Example of a kymograph generated using the reslice function on ImageJ program, showing the distance and time in the *y* and *x* axis, respectively. A white box is used to highlight an actin protrusion and the image magnified in (**f**). (**f**) Example of a measurement of actin protrusion distance, D, persistence, P and protrusion rate, R. (**g**) Kymograph measurements obtained for protrusion distance, ** *p* ≤ 0.01 (**h**) persistence and (**i**) protrusion rate.

To identify cellular and biochemical alterations of the MDA-MB-231 cells with mutations in MRCK genes, we investigated the breast cancer cells in 2D culture. MRCK single and double KO cells were morphologically similar to control MDA-MB-231, with a slightly elongated cell shape (Figure 6a). Assessing proliferation in a confluency assay, KO lines and control cells showed a similar growth rate (Figure 6b). Furthermore, adhesion to collagen was identical in all cells (Figure 6c), suggesting that integrin mediated collagen binding is not changed by loss of MRCK.

To study dynamic changes in F-actin formation and protrusions at the plasma membrane, we transfected the cells with the fluorescent F-actin marker LifeAct and monitored F-actin by time lapse fluorescence microscopy. In randomly chosen sections through lamellipodial regions, distance, persistence and rate of protrusions was measured by kymograph analysis (Figure 6d–f). Distance of protrusions was slightly higher in MRCKα KO and lower in DKO, suggesting that loss of MRCKα and MRCKβ results in subtle changes in actin cytoskeleton dynamics (Figure 6g–i).

**Figure 7:**
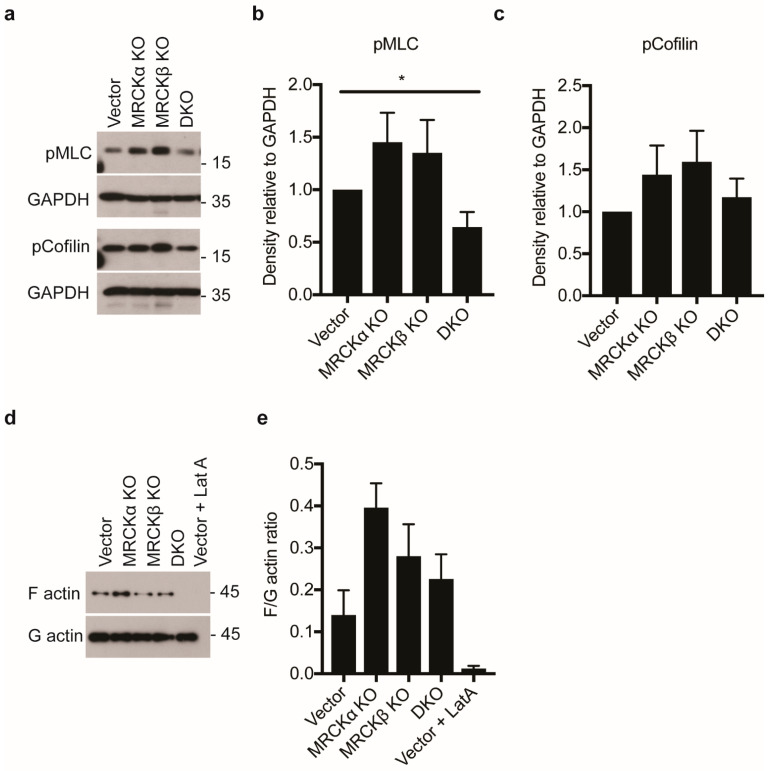
MRCK KO does not effectively reduce myosin and cofilin phosphorylation in MDA-MB-231 cells (**a**) Representative immunoblot showing myosin light chain (pMLC) and phospho-cofilin in MDA-MB-231 cells with MRCKα KO, MRCKβ KO, or DKO. (**b**,**c**) Quantitation of indicated proteins from lysates obtained from the knockout cells (*n*: 6/6/6/6; mean ± SEM, one-way ANOVA; * *p* ≤ 0.05). (**d**) Representative immunoblot showing levels of F-actin and G-actin in control, MRCKα KO, MRCKβ KO and DKO MDA-MB-231 cells. As a control, cells were also treated with 1 μM latrunculin A (Lat A) for 15 min. (**e**) Quantification of F/G-actin ratio (*n*: 6/6/6/6; mean ± SEM, not significant with one-way ANOVA, * *p* ≤ 0.05 with two-tailed *t*-test for Vector vs. MRCKa KO).

Earlier, MRCKα and MRCKβ were shown to regulate together with ROCK the phosphorylation of cofilin and myosin light chain (MLC), corresponding to increased F-actin formation and increased cell contraction. Unexpectedly, in our MDA-MB-231 cells neither in MRCKα KO nor in MRCKβ KO a decrease in pMLC, pCofilin, or F-actin was detectable (Figure 7a–e). They rather appeared to be increased in MRCKα KO MDA-MB-231 cells, although the differences were not significant. DKO cells showed slightly reduced pMLC compared to vector transfected controls.

We then tested for differential gene expression by RNA sequencing, excluding poorly expressed genes. Functional annotation of the differentially regulated genes by DAVID suggested a coordinated regulation of immune response related genes in the DKO and the MRCKα KO cells, with no obvious indication of any alterations in pathways related to cell migration (Supplementary Figures S3–S5). The lists of top up- and down-regulated genes in the MRCK KO cells can be found as Supplementary Tables S1–S6). Within the lists, none of the genes altered more than 2-fold was obviously related to actin cytoskeletal regulation or cell migration, besides the actin nucleator LMOD1 [24], which was upregulated in MRCKβ KO cells, but neither in MRCKα KO nor in DKO cells. This suggests that MRCKα and MRCKβ regulate cell migration mainly at posttranscriptional level. Taken together, MRCKα KO and DKO cells display small changes in 2D culture compared to control cells.

### 3.6. Collective 3D Invasion of 4T1 Breast Cancer Is Not Dependent on MRCKα 

**Figure 8:**
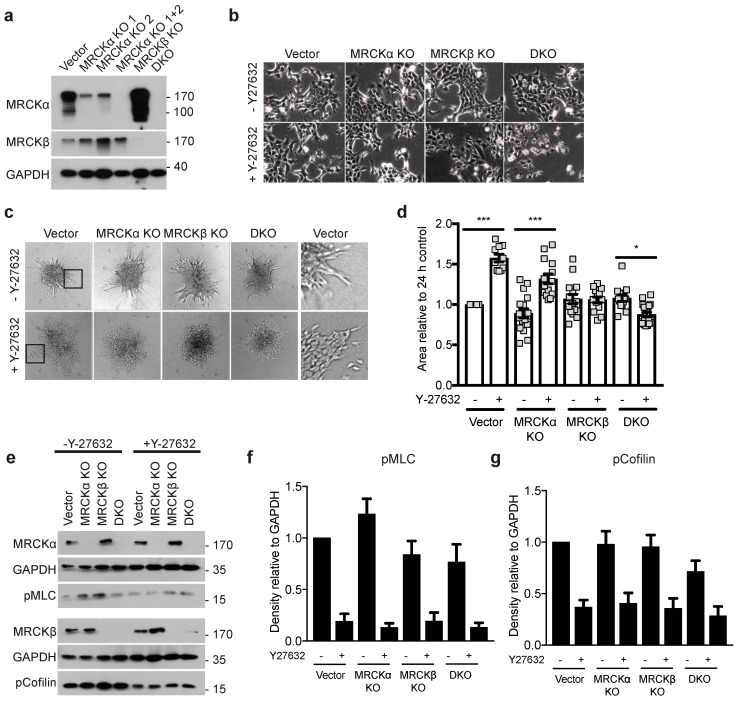
MRCKα and MRCKβ are dispensable in 4T1 cells matrix invasion. (**a**) Representative immunoblot showing successful knockout of MRCKα and MRCKβ in 4T1 cells. DKO was achieved by using one of two different CRISPR gRNAs for MRCKα (“MRCKα 1 + 2”) and one gRNA for MRCKβ (**b**) Representative phase contrast images of control, MRCKα, MRCKβ and DKO 4T1 cell morphologies in the presence and absence of Y-27632 (10 μM). (**c**) 3D matrix invasion of MRCK KO 4T1 spheroids in type I collagen, in the presence and absence of Y-27632 (10 μM). Black boxes highlight the invasion front of the control spheroids, as shown magnified in the right panel. (**d**) Quantification of the area of invasion of the 4T1 spheroids from (**c**) relative to 24 h control (*n*: 3; mean ± SEM, one-way ANOVA; * *p* ≤ 0.05, *** *p* ≤ 0.001). (**e**) Representative immunoblot and (**f**,**g**) quantification of the expression of the indicated proteins. (*n*: 5; mean ± SEM, one-way ANOVA).

MDA-MB-231 cells invade collagen gels mostly as single cells, while the tumor organoids of MRCKα KO and control breast cancers only showed collective migration (Supplementary Figure S1b). To test the function of MRCKα and MRCKβ in 3D collagen gel invasion assays also with triple-negative breast cancer cells showing collective migration, we performed additional experiments with murine 4T1 cells. CRISPR genome editing resulted in efficient deletion of MRCKα and MRCKβ protein as tested by Western blot (Figure 8a). As was the case for MDA-MB-231 cells, MRCKα KO cells have increased MRCKβ expression, although the reverse was not consistently observed (Supplementary Figure S6a,b). Control, KO and DKO cells showed similar morphology and were both growing in colonies, suggesting strong cell-cell interactions (Figure 8b). In 3D collagen gel invasion assays, 4T1 cells displayed collective migration similar to the organoids isolated form the MMTV-PyMT mice (Figure 8c,d). However, neither deletion of MRCKα or MRCKβ or both reduced the invasion significantly. 

Previously, it had been shown that inhibition of ROCK1 and ROCK2, in addition to MRCKα and MRCKβ strongly impaired migration of cancer cells [9,10]. We therefore repeated the collagen invasion assay in the presence of Y-27632, which efficiently inhibits ROCK1 and ROCK2. Unexpectedly, however, Y-27632 increased the collagen invasion of 4T1 cells (Figure 8c,d). Deletion of MRCKα and even more of MRCKβ blocked this increased invasion. DKO cells showed a reduced invasion when ROCK was inhibited. In the presence of Y-27632, cell-cell borders became clearly visible in all 4T1 lines in 3D, suggesting altered cell-cell contacts (Figure 8c). Still, cells appeared not to migrate as individual cells in contrast to MDA-MB-231 (Figure 5). In 2D, Y-27632 treated cells grew less in colonies and displayed thin and long plasma membrane protrusions (Figure 8b). Phosphorylated MLC (pMLC) and pCofilin were not significantly affected by the loss of MRCKα, MRCKβ, or both (Figure 8e–g). These data suggest that inhibition of ROCK signaling promotes invasion of 4T1 breast cancer cells in an MRCK dependent manner, but a dispensable role for MRCKα and MRCKβ in 4T1 cell for collective cell migration in 3D. 

### 3.7. MRCKα Is Co-Amplified with the Oncogenes AKT3 and ARID4B in Breast Cancer

**Figure 9:**
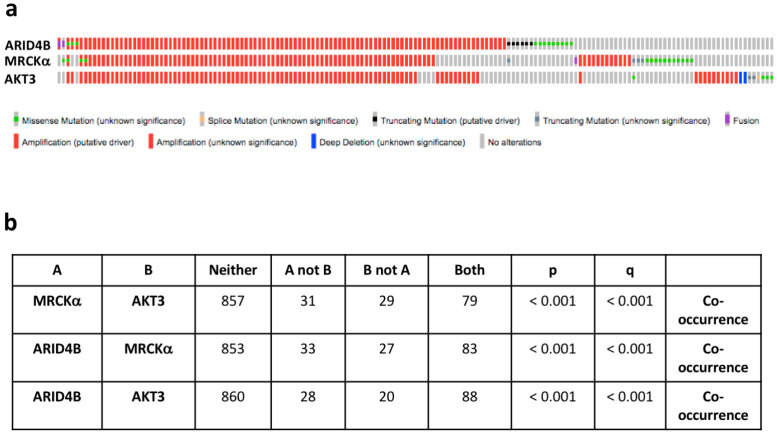
MRCKα is co-amplified with ARID4B and AKT3. (**a**) Oncoprints of genomic alterations of MRCKα, ARID4B and AKT3 in human breast cancers. (**b**) Analysis of co-occurrence of MRCKα with the oncogenes ARID4B and AKT3. All analyses were generated in cBioportal (TCGA PanCancer Atlas, breast cancer samples, accessed on 20 February 2021).

In cancer, some genes are amplified not because they are driver genes themselves but because they are co-amplified with an oncogene in the vicinity. We therefore, tested in breast cancers of the TCGA PanCancer dataset whether other amplified oncogenes are close to the chromosomal location of the MRCKα gene (Chr 1q41.13). Indeed, both ARID4B (Chr 1q42.3) and AKT3 (Chr 1q43-q44) are in the neighborhood of the MRCKα gene. ARID4B is an activator of the PI3K-AKT pathway, an oncogene in PTEN-deficient prostate cancer [25] and amplified in 12% of breast cancers in the TCGA data set. AKT3 showed an oncogenic effect in the murine MMTV-PymT mammary cancer model [26]. Around 11% of human breast cancers show amplification of AKT3. Interestingly, ARID4B, MRCKα and AKT3 are mostly amplified together and show a highly significant co-occurrence of genetic alterations in breast cancer (Figure 9a,b).

Importantly, patients with amplified ARID4B showed a significant decrease in disease-free survival (*p* = 0.049), in contrast to patients with amplified MRCKα (*p* = 0.101) and AKT3 (*p* = 0.108), despite the high overlap of the patient populations. Cancer with amplified ARID4B displayed a 29% increase in ARID4B expression, which was highly significant (1.17 × 10^−8^). AKT3 expression was similarly increased in cancers with AKT3 amplification, but with a much lower significance (28%; *p* = 0.036). Compared to amplifications of MRCKα and AKT3, amplifications of ARID4B showed the strongest enrichment in the Basal cancer subtype.

These statistical analyses of patient data suggest that ARID4B might have the strongest tumor promoting effect of the three co-amplified genes. Furthermore, the correlation of MRCKα expression with breast cancer formation and progression appears to be at least partly due to the co-amplification with AKT3 and in particular ARID4B. 

## 4. Discussion

In order to study MRCKα function in breast cancer, we first established mice with a constitutive inactivation of the MRCKα gene in all cells. Previous in vitro studies suggested a role for MRCKα in developmental processes involving Cdc42 dependent cell migration and in neurite formation in the brain [12,27]. Analysis of our MRCKα KO mice did not reveal an obvious developmental function, which might be caused by redundant function of the closely related MRCKβ gene.

Despite the genetic amplification of MRCKα in human breast cancers, we found that loss of MRCKα had no obvious effect on primary tumor growth in the murine MMTV-PyMT model. Since inhibition of MRCKα and MRCKβ decreased papilloma volume in a DMBA/TPA induced skin tumor model [4], functional redundancy between MRCKα and MRCKβ or tumor specific function could explain the normal growth of MRCKα KO breast cancers. Cell-type specific effect of MRCKs were already observed earlier when testing 757 cancer cell lines from 45 different tumors for their sensitivity to MRCK inhibitors [4]. Here, hematopoietic cancers showed growth inhibition at much lower doses than breast cancer cell lines. In this context, it should be noted that acute inhibition by small molecule inhibitors might result in different effects than chronic elimination of a protein, which could trigger compensatory mechanisms [28]. We tested for a compensatory effect of ROCK in 4T1 cells lacking MRCKα and MRCKβ by treatment with the ROCK inhibitor Y27632, but did not observe it in this cell line. 

MDA-MB-231 and 4T1 are triple negative breast cancer cell lines, which is similar to the Basal breast cell cancer subtype. The MMTV-PyMT breast cancer corresponds to the human Luminal B subtype [23]. However, more extensive experiments are required to test whether the findings can be extrapolated to all breast cancer subtypes.

An alternative explanation for the reported results is that MRCKα is a passenger gene that is co-amplified with ARID4B and AKT3, which have been reported to be driver genes in earlier studies, although no clinical studies have been carried out investigating their prognostic value. High expression of MRCKα could therefore correlate with a bad prognosis, although MRCKα itself might not be an oncogene.

Based on earlier studies, a role for MRCKα was expected in invasion and metastasis of breast cancer cells. Previously, it was shown that siRNA mediated inhibition of MRCKα and MRCKβ in cancer cells reduced their invasion into collagen gel [10]. Similarly, simultaneous knockdown of MRCKα and MRCKβ in MDA-MB-231 cells decreased their invasion into matrigel [9]. Moreover, collective migration of squamous cell carcinoma cells SSC12 in an organotypic culture system including fibroblasts was strongly reduced after knockdown of MRCKα and MRCKβ [11]. In human breast cancer, MRCKα is amplified in many patients, correlating with early metastasis [13]. Indeed, we found that deleting the MRCKα gene either alone or together with MRCKβ significantly reduced 3D collagen invasion of MDA-MB-231 cells. This altered invasion in 3D did not correlate with changes in pMLC or pCofilin in 2D culture, indicating either a different regulation of cell contraction (pMLC) and F-actin severing (pCofilin) in 3D vs. 2D or a different mechanism for MRCKα dependent invasion of MDA-MB-231 cells.

In the MMTV-PyMT breast cancer model, however, no significant reduction of lung metastases was observed in the absence of MRCKα. In contrast to MDA-MB-231 cells, cancer cells of the MMTV-PyMT model showed collective migration in collagen as tested with tumor organoids, suggesting that the mode of migration might determine the importance of MRCKα in breast cancer. This notion is supported by the finding that MRCKα is not required for the 3D collagen invasion of the murine 4T1 breast cancer cell line, which also shows collective migration. Whether species specific differences contribute to the observed changes could not be determined.

Using a murine knockout model, the current study reveals that MRCKα is not essential for PyMT induced breast cancer. However, our results do not exclude that increased expression of MRCKα could promote breast cancer or exacerbate its progression.

Taken together, these data support further analysis of MRCKα expression as a possible prognostic marker, but do not validate MRCKα as a drug target in breast cancer, although it might play a role in a subset of cancers showing mesenchymal migration.

## Data Availability

The data that supports the findings of this study are available in the figures and the supplementary material of this article. RNAseq data are available at the Gene Expression Omnibus (GEO) database (https://www.ncbi.nlm.nih.gov/geo/, under the accession number GSE169000.

## Supplementary Materials

The following are available online at https://www.mdpi.com/article/10.3390/cells10040942/s1. Supplementary legends. Figure S1: (a) Overall survival of breast cancer patients with high and low expression of MRCKβ (*p* = 0.899). (e) Breast cancer subtype distribution of patients with high and low expression of MRCKβ. Graphs were generated in cBioPortal.org using the TCGA PanCancer Atlas data set for invasive breast carcinoma (accessed on 20 March 2021). Figure S2: MRCKα knockout tumour organoids metastasize normally, Figure S3. RNA-seq analysis of MRCKα knockout cells, Figure S4. RNA-seq analysis of MRCKβ knockout cells, Figure S5. RNA-seq analysis of MRCK DKO cell, Figure S6. MRCKα knockout in 4T1 cells results in MRCKβ up-regulation, Video S1: Invasion of control MDA-MB-231 breast cancer cells into 3D collagen gel, Video S2: Invasion of MRCKα/MRCKβ DKO MDA-MB-231 breast cancer cells into 3D collagen gel.
