# Supplementary material for: MRCKα Is Dispensable for Breast Cancer Development in the MMTV-PyMT Model
Source: Cells. 2021 Apr 19;10(4):942. doi: 10.3390/cells10040942 (PMC8073694; doi:10.3390/cells10040942)
Supplement: Supplementary file 1 [file cells-10-00942-s001.zip › pdf supp/Supp tables 1 - 6.pdf]

Suppl. Table 1. Top 20 genes up-regulated in MDA231 MRCKα KO cells

Symbol	Log2 FC	Protein name
**TLL2**	4.58	tolloid like 2
**FAM221B**	3.53	family with sequence similarity 221 member B
**LYPD6B**	3.40	LY6/PLAUR domain containing 6B
**LOC101927262**	3.28	melanoma-associated antigen C1-like
**UNC5B**	3.17	unc-5 netrin receptor B
**AADAC**	3.09	arylacetamide deacetylase
**SIX3**	2.93	SIX homeobox 3
**MTAP**	2.86	methylthioadenosine phosphorylase
**LTB**	2.83	lymphotoxin beta
**TEX19**	2.81	testis expressed 19
**NOD2**	2.68	nucleotide binding oligomerization domain containing 2
**ZNF396**	2.61	zinc finger protein 396
**SPATA31D4**	2.58	SPATA31 subfamily D member 4
**SPOCK2**	2.58	SPARC/osteonectin, cwcv and kazal like domains proteoglycan 2
**TNR**	2.58	tenascin R
**SLC25A18**	2.43	solute carrier family 25 member 18
**ENTHD1**	2.39	ENTH domain containing 1
**CCL17**	2.32	C-C motif chemokine ligand 17
**GSX2**	2.32	GS homeobox 2
**MAFA**	2.32	MAF bZIP transcription factor A

Suppl. Table 2. Top 20 genes down-regulated in MDA231 MRCKα KO cells

Symbol	Log2 FC	Protein name
**TEX101**	-4.66	testis expressed 101
**PRR23C**	-3.54	proline rich 23C
**CASP14**	-3.52	caspase 14
**PKNOX2**	-3.46	PBX/knotted 1 homeobox 2
**SSX5**	-3.16	SSX family member 5
**OXCT2**	-3.07	3-oxoacid CoA-transferase 2
**GOLGA8H**	-3.01	golgin A8 family member H
**ADARB2**	-3.00	adenosine deaminase, RNA specific B2 (inactive)
**LOC107987477**	-2.96	uncharacterized LOC107987477
**NTSR1**	-2.91	neurotensin receptor 1
**FANCD2P2**	-2.89	Fanconi anemia complementation group D2 pseudogene 2
**RIMBP3B**	-2.86	RIMS binding protein 3B
**FNTB**	-2.77	farnesyltransferase, CAAX box, beta
**ACTG2**	-2.77	actin, gamma 2, smooth muscle, enteric
**LRRC15**	-2.75	leucine rich repeat containing 15
**ACOT11**	-2.72	acyl-CoA thioesterase 11
**PYY**	-2.71	peptide YY
**ADGRF4**	-2.70	adhesion G protein-coupled receptor F4
**LOC107987253**	-2.58	uncharacterized protein FAM215A-like
**LOC107987254**	-2.58	uncharacterized protein FAM215A-like

Suppl. Table 3. Top 20 genes up-regulated in MDA231 MRCKβ KO cells

Symbol	Log2 FC	Protein name
**LMOD1**	3.70	leiomodin 1
**TLL2**	3.46	tolloid like 2
**SLFN11**	2.81	schlafen family member 11
**LOC107984156**	2.75	ADP-ribosylation factor-like protein 17
**SYT7**	2.58	synaptotagmin 7
**COL11A2**	2.58	collagen type XI alpha 2 chain
**FAM221B**	2.55	family with sequence similarity 221 member B
**ZNF382**	2.53	zinc finger protein 382
**LTB**	2.50	lymphotoxin beta
**AADAC**	2.46	arylacetamide deacetylase
**NIPSNAP3B**	2.46	nipsnap homolog 3B
**DENND6B**	2.32	DENN domain containing 6B
**FAM167B**	2.32	family with sequence similarity 167 member B
**BTBD8**	2.31	BTB domain containing 8
**SKAP1**	2.31	src kinase associated phosphoprotein 1
**INSC**	2.28	inscuteable homolog (Drosophila)
**TRPM2**	2.24	transient receptor potential cation channel subfamily M member 2
**AGT**	2.18	angiotensinogen
**PPP1R32**	2.14	protein phosphatase 1 regulatory subunit 32
**PIK3IP1**	2.12	phosphoinositide-3-kinase interacting protein 1

Suppl. Table 4. Top 20 genes down-regulated in MDA231 MRCKβ KO cells

Symbol	Log2 FC	Protein name
**SSX5**	-3.30	SSX family member 5
**CYBB**	-3.21	cytochrome b-245 beta chain
**NR5A2**	-3.04	nuclear receptor subfamily 5 group A member 2
**CTSW**	-2.97	cathepsin W
**PRY**	-2.91	PTPN13-like, Y-linked
**TMEM233**	-2.84	transmembrane protein 233
**UPK1B**	-2.83	uroplakin 1B
**C9orf66**	-2.75	chromosome 9 open reading frame 66
**EME2**	-2.75	essential meiotic structure-specific endonuclease subunit 2
**ADGRF4**	-2.70	adhesion G protein-coupled receptor F4
**LOC105379282**	-2.70	glucose-dependent insulinotropic receptor-like
**LOC100130357**	-2.70	uncharacterized LOC100130357
**IL10**	-2.62	interleukin 10
**PROCA1**	-2.60	protein interacting with cyclin A1
**SMKR1**	-2.60	small lysine rich protein 1
**GLIPR1L1**	-2.56	GLI pathogenesis related 1 like 1
**PIANP**	-2.54	PILR alpha associated neural protein
**TRIM17**	-2.54	tripartite motif containing 17
**BTC**	-2.50	betacellulin
**RNF43**	-2.50	ring finger protein 43

Suppl. Table 5. Top 20 genes up-regulated in MDA231 DKO cells

Symbol	Log2 FC	Protein name
**LTB**	3.63	lymphotoxin beta
**FAM221B**	2.97	family with sequence similarity 221 member B
**EGR1**	2.49	early growth response 1
**SYT7**	2.35	synaptotagmin 7
**ARC**	2.30	activity regulated cytoskeleton associated protein
**ALOXE3**	2.11	arachidonate lipoxygenase 3
**NIPSNAP3B**	2.09	nipsnap homolog 3B
**SLC12A5**	2.09	solute carrier family 12 member 5
**SLC25A18**	2.07	solute carrier family 25 member 18
**C5orf64**	2.04	chromosome 5 open reading frame 64
**CALCRL**	2.00	calcitonin receptor like receptor
**GCNT4**	1.93	glucosaminyl (N-acetyl) transferase 4, core 2
**LTF**	1.90	lactotransferrin
**TRPM2**	1.90	transient receptor potential cation channel subfamily M member 2
**CXCL11**	1.81	C-X-C motif chemokine ligand 11
**MUSTN1**	1.75	musculoskeletal, embryonic nuclear protein 1
**TNFAIP3**	1.73	TNF alpha induced protein 3
**TRPM6**	1.72	transient receptor potential cation channel subfamily M member 6
**DHRS2**	1.70	dehydrogenase/reductase 2
**CSF2RB**	1.67	colony stimulating factor 2 receptor beta common subunit

Suppl. Table 6. Top 20 genes down-regulated in MDA231 DKO cells

Symbol	Log2 FC	Protein name
**NR5A2**	-2.72	nuclear receptor subfamily 5 group A member 2
**PYY**	-2.56	peptide YY
**RUNDC3A**	-2.32	RUN domain containing 3A
**GNAT1**	-2.28	G protein subunit alpha transducin 1
**CRLF2**	-2.15	cytokine receptor-like factor 2
**GDPGP1**	-2.13	GDP-D-glucose phosphorylase 1
**C9orf66**	-2.11	chromosome 9 open reading frame 66
**GHR**	-2.07	growth hormone receptor
**KIAA2012**	-1.95	KIAA2012
**PRY**	-1.91	PTPN13-like, Y-linked
**PCSK6**	-1.91	proprotein convertase subtilisin/kexin type 6
**AMER3**	-1.87	APC membrane recruitment protein 3
**GNRH1**	-1.83	gonadotropin releasing hormone 1
**MYO15B**	-1.78	myosin XVB
**ZNF474**	-1.77	zinc finger protein 474
**TMEM37**	-1.75	transmembrane protein 37
**SSX5**	-1.74	SSX family member 5
**CRLF1**	-1.68	cytokine receptor like factor 1
**HLA-G**	-1.64	major histocompatibility complex, class I, G
**HIGD1B**	-1.62	HIG1 hypoxia inducible domain family member 1B
