# Supplementary figures and images for: MRCKα Is Dispensable for Breast Cancer Development in the MMTV-PyMT Model
Source: Cells. 2021 Apr 19;10(4):942. doi: 10.3390/cells10040942 (PMC8073694; doi:10.3390/cells10040942)

# Supp. Figure S1

a

MRCK $\beta$  mRNA

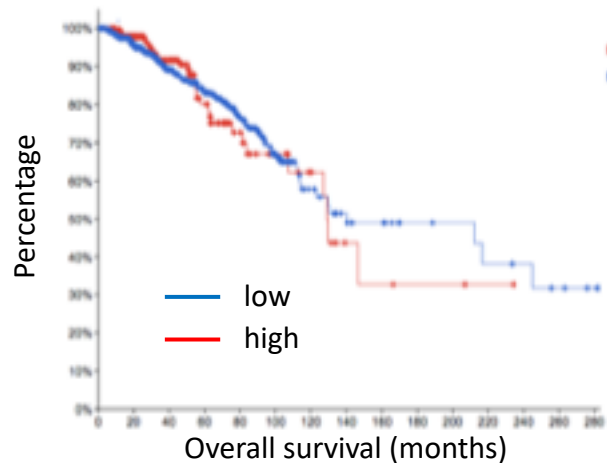

b

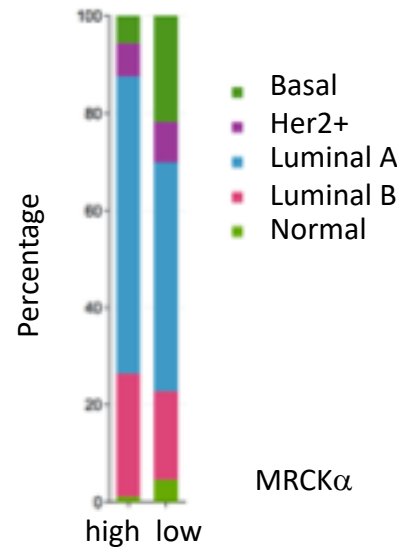

Supplement: Supplementary file 1 [file cells-10-00942-s001.zip › pdf supp/Supp fig 1.pdf]

Supplementary Figure 2

**a**

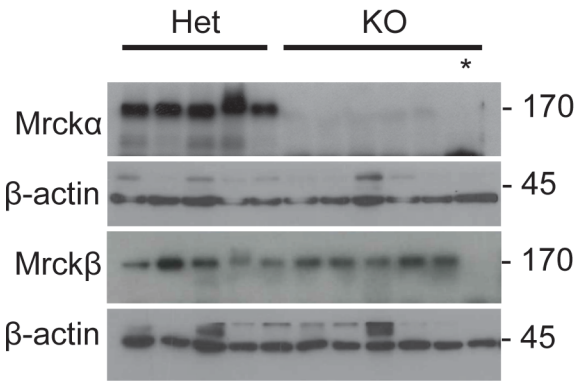

**b**

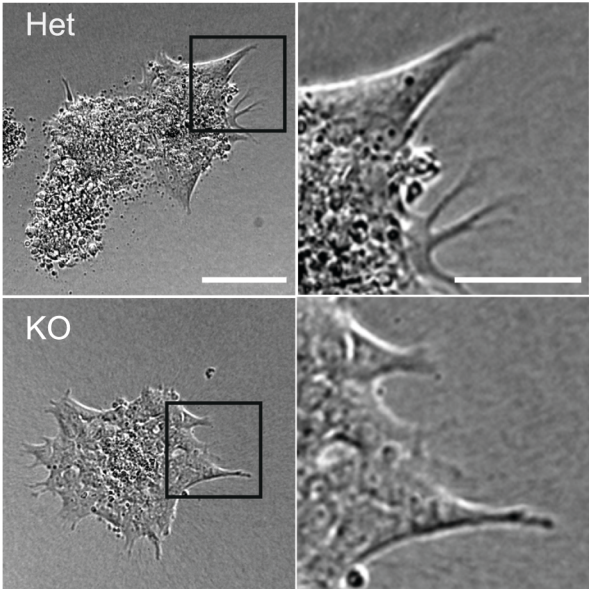

Supplement: Supplementary file 1 [file cells-10-00942-s001.zip › pdf supp/Supp fig 2.pdf]

Supplementary Figure 3

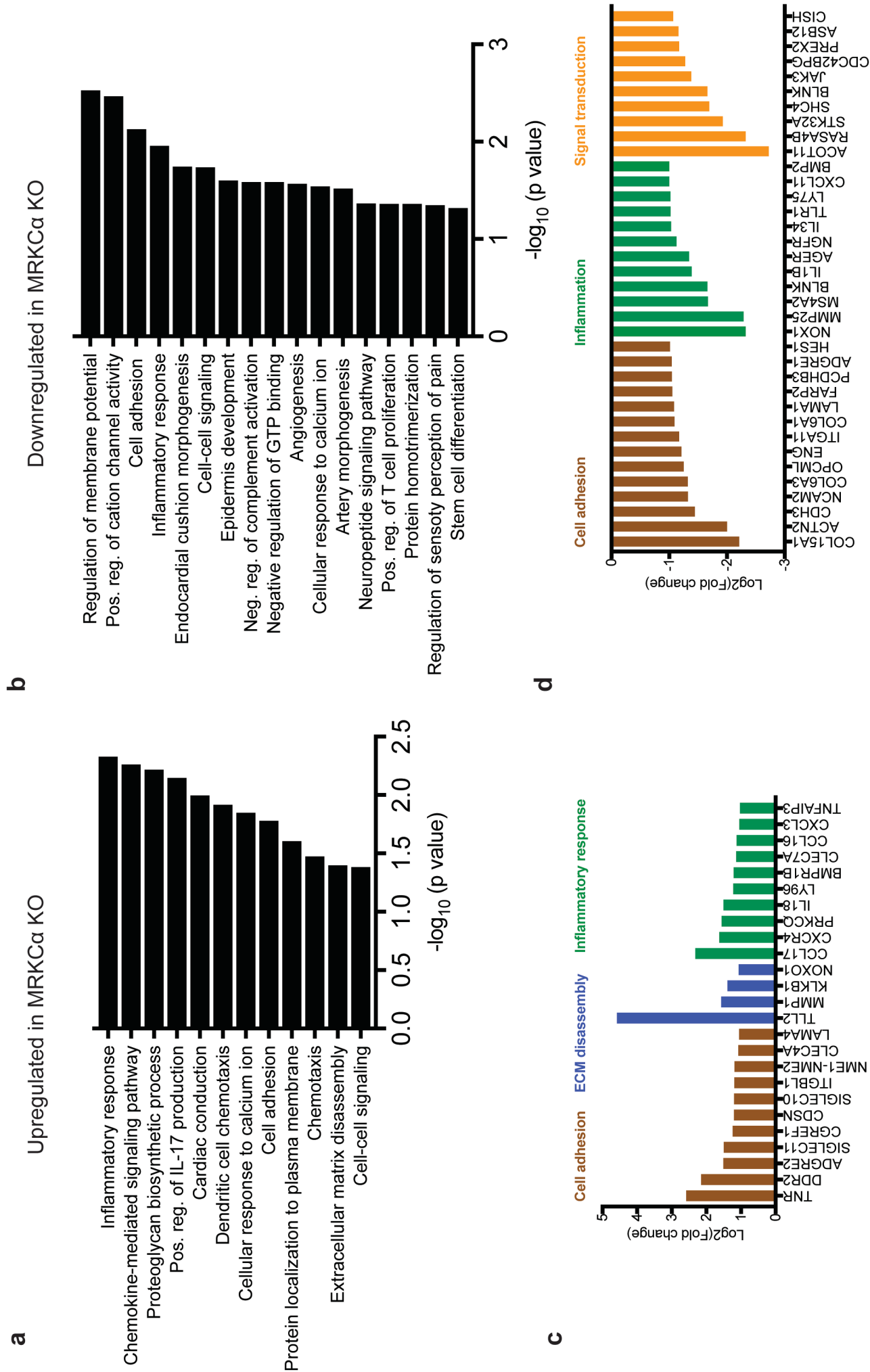

Supplement: Supplementary file 1 [file cells-10-00942-s001.zip › pdf supp/Supp fig 3.pdf]

Supplementary Figure 4

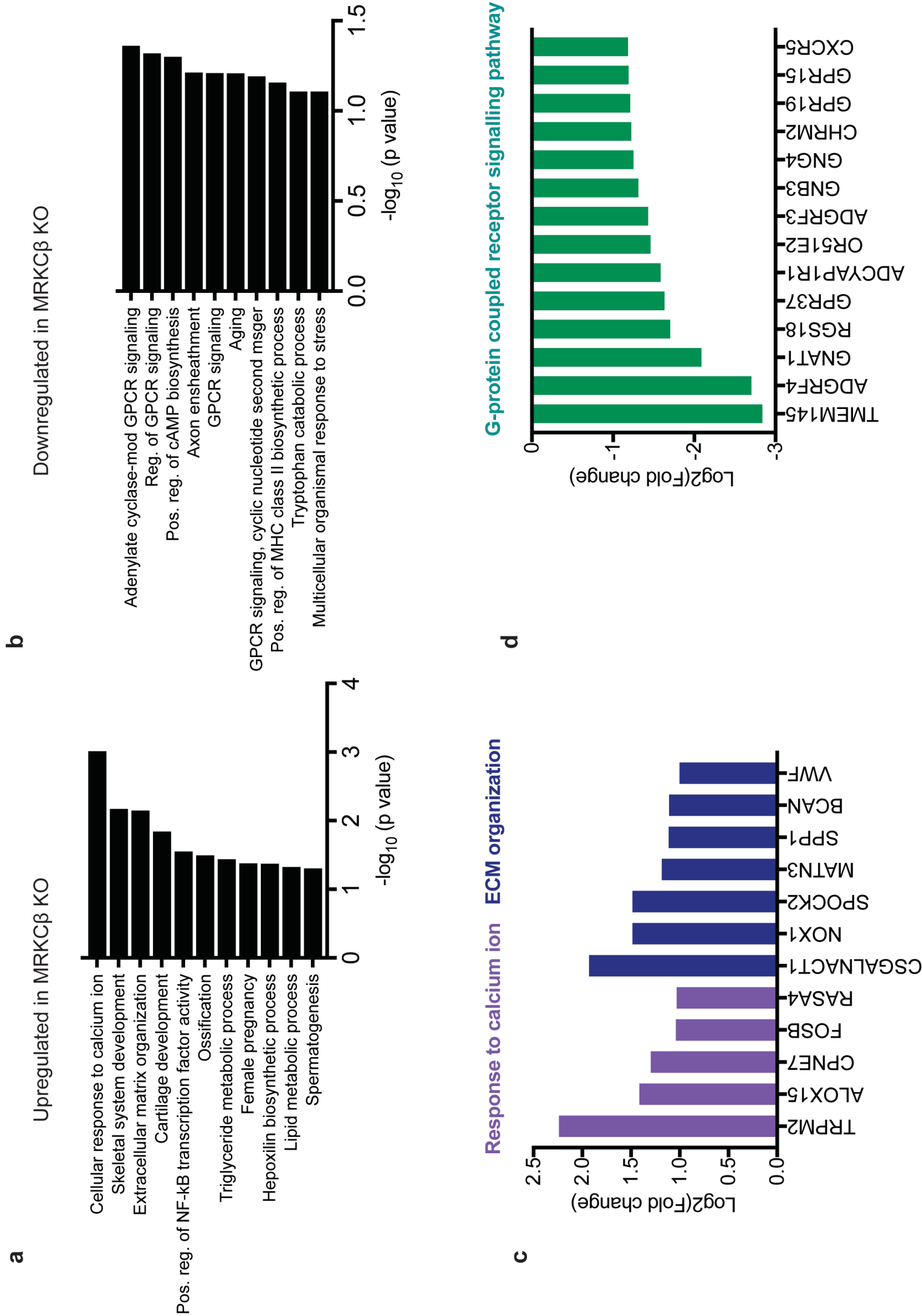

Supplement: Supplementary file 1 [file cells-10-00942-s001.zip › pdf supp/Supp fig 4.pdf]

Supplementary Figure 5

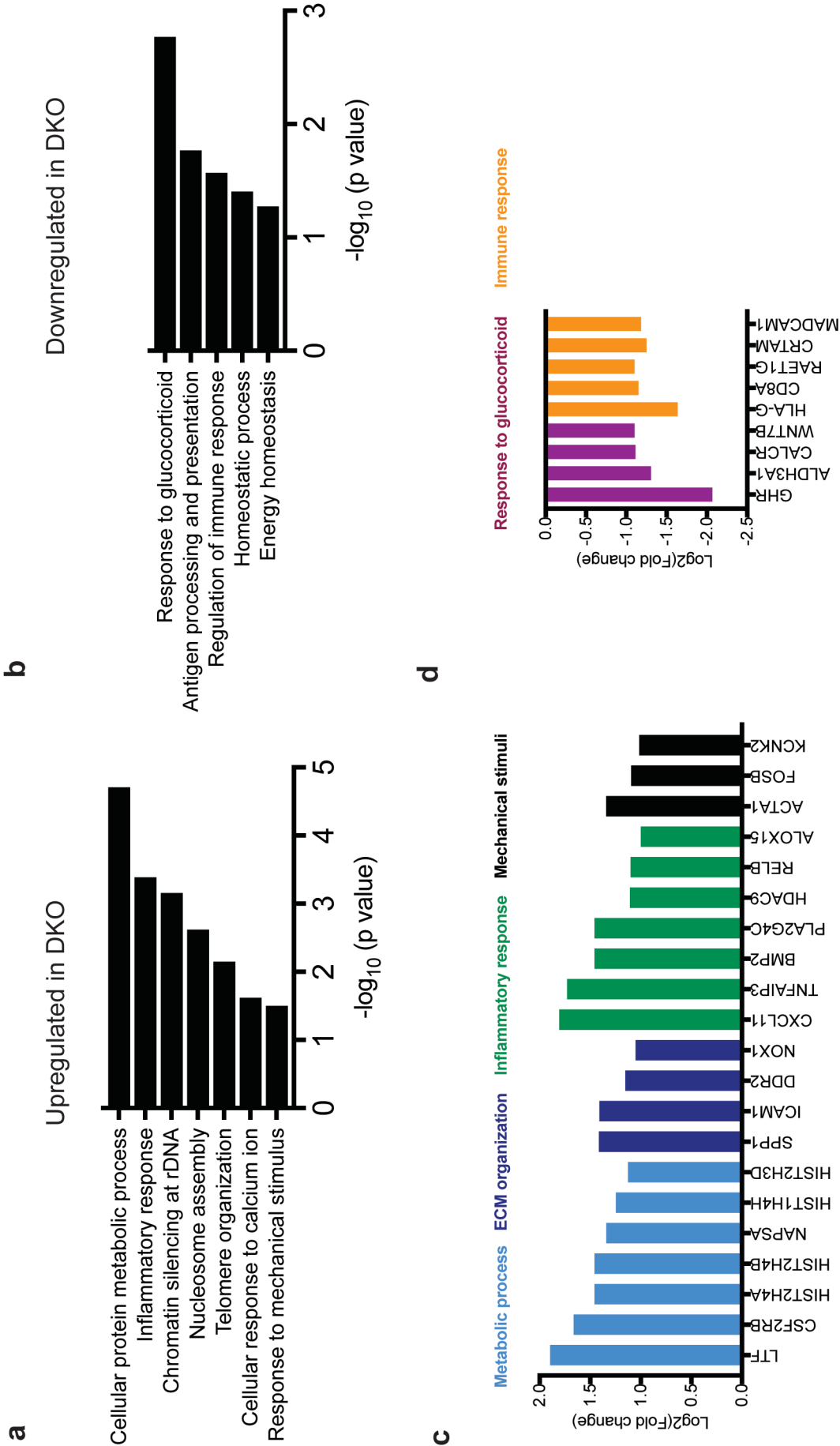

Supplement: Supplementary file 1 [file cells-10-00942-s001.zip › pdf supp/Supp fig 5.pdf]

Supplementary Figure 6

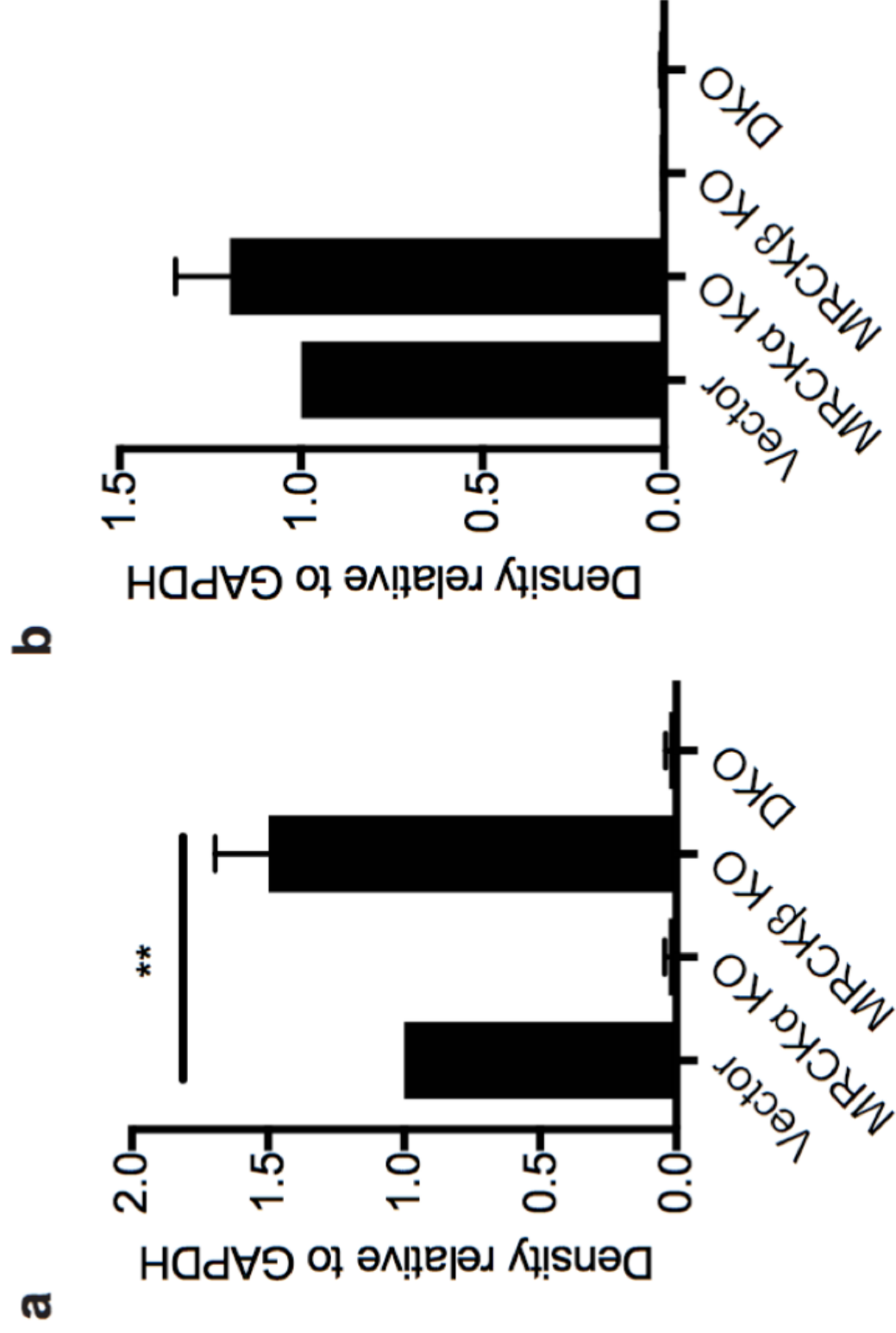

Supplement: Supplementary file 1 [file cells-10-00942-s001.zip › pdf supp/Supp fig 6.pdf]
